# Exploiting Long Non-Coding RNAs and Circular RNAs as Pharmacological Targets in Triple-Negative Breast Cancer Treatment

**DOI:** 10.3390/cancers15164181

**Published:** 2023-08-20

**Authors:** Alina Catalina Palcau, Renata Brandi, Nikolay Hristov Mehterov, Claudio Botti, Giovanni Blandino, Claudio Pulito

**Affiliations:** 1 Translational Oncology Research Unit, Department of Research, Advanced Diagnostic and Technological Innovation, IRCCS Regina Elena National Cancer Institute, 00144 Rome, Italy; alina.palcau@ifo.it (A.C.P.); renata.brandi@ifo.it (R.B.); giovanni.blandino@ifo.it (G.B.); 2 Department of Medical Biology, Medical University-Plovdiv, 4002 Plovdiv, Bulgaria; nikolay.mehterov@mu-plovdiv.bg; 3 Research Institute, Medical University-Plovdiv, 4002 Plovdiv, Bulgaria; 4 Breast Surgery Unit, IRCCS Regina Elena National Cancer Institute, 00144 Rome, Italy; claudio.botti@ifo.it

**Keywords:** long non-coding RNA, circular RNA, triple-negative breast cancer, non-coding RNA, biomarker

## Abstract

**Simple Summary:**

Triple-negative breast cancer accounts for 15–20% of breast tumors. It is a very aggressive and heterogeneous disease characterized by the absence of druggable molecular targets. In that context, understanding the role of non-coding RNAs and their implication in tumorigenesis could represent an opportunity for the development of new therapeutic strategies, as well as for the identification of reliable prognostic biomarkers. Recurrence and development of drugs-resistance represents the most challenging aspects in triple-negative breast cancer. Non-coding RNAs’ unique characteristics make them reliable biomarkers for monitoring cancer treatment, potentially able to identify recurrence or chemoresistance.

**Abstract:**

Breast cancer is one of the most frequent causes of cancer death among women worldwide. In particular, triple-negative breast cancer (TNBC) represents the most aggressive breast cancer subtype because it is characterized by the absence of molecular targets, thus making it an orphan type of malignancy. The discovery of new molecular druggable targets is mandatory to improve treatment success. In that context, non-coding RNAs represent an opportunity for modulation of cancer. They are RNA molecules with apparently no protein coding potential, which have been already demonstrated to play pivotal roles within cells, being involved in different processes, such as proliferation, cell cycle regulation, apoptosis, migration, and diseases, including cancer. Accordingly, they could be used as targets for future TNBC personalized therapy. Moreover, the peculiar characteristics of non-coding RNAs make them reliable biomarkers to monitor cancer treatment, thus, to monitor recurrence or chemoresistance, which are the most challenging aspects in TNBC. In the present review, we focused on the oncogenic or oncosuppressor role of long non-coding RNAs (lncRNAs) and circular RNAs (circRNAs) mostly involved in TNBC, highlighting their mode of action and depicting their potential role as a biomarker and/or as targets of new non-coding RNA-based therapeutics.

## 1. Introduction

Breast cancer is one of the most frequent cancers leading to deaths in women worldwide [1]. It is a heterogenous type of cancer that, from a molecular point of view, can be classified into four different subtypes, according to the expression of three pivotal receptors: estrogen receptor (ER), progesterone receptor (PR), and human epidermal growth factor receptor 2 (HER2). Accordingly, breast cancer subtypes are Luminal A, Luminal B, HER2, and triple-negative breast cancer (TNBC).

TNBC is the most aggressive breast cancer subtype because it lacks the expression of all ER, PR, and HER2 receptors. It presents high intra-heterogeneity; indeed, four different subtypes can be identified: basal-like immune-activated, basal-like immunosuppressed, mesenchymal, and luminal androgen receptor [2].

Moreover, it is often associated with hereditary conditions, such as BRCA1 and BRCA2 mutations. Other mutations in key DNA repair genes or oncosuppressors, such as p53, are common, as well as upregulation of specific genes and their related pathways involved in cell proliferation, migration, and inhibition of apoptosis [3,4].

Epigenetically, TNBC is also characterized by a chromatin hypomethylation pattern, which supports genome instability. Indeed, this breast cancer subtype is characterized by a shorter median time to relapse. Additionally, TNBC is associated with a high risk of metastases, leading to a poor overall survival rate. Chemotherapy and radiotherapy are the gold standard treatments since there are no molecular targets that can be exploited for TNBC treatment [5,6]. In particular, current treatment strategies include the use of taxanes, platinum agents, anthracyclines, and ixabepilone. Unfortunately, drug resistance, as well as the lack of druggable targets, represent the major issues that need to be overcome. In that scenario, non-coding RNAs could represent an opportunity for solving the above-raised issues.

**Figure 1:**
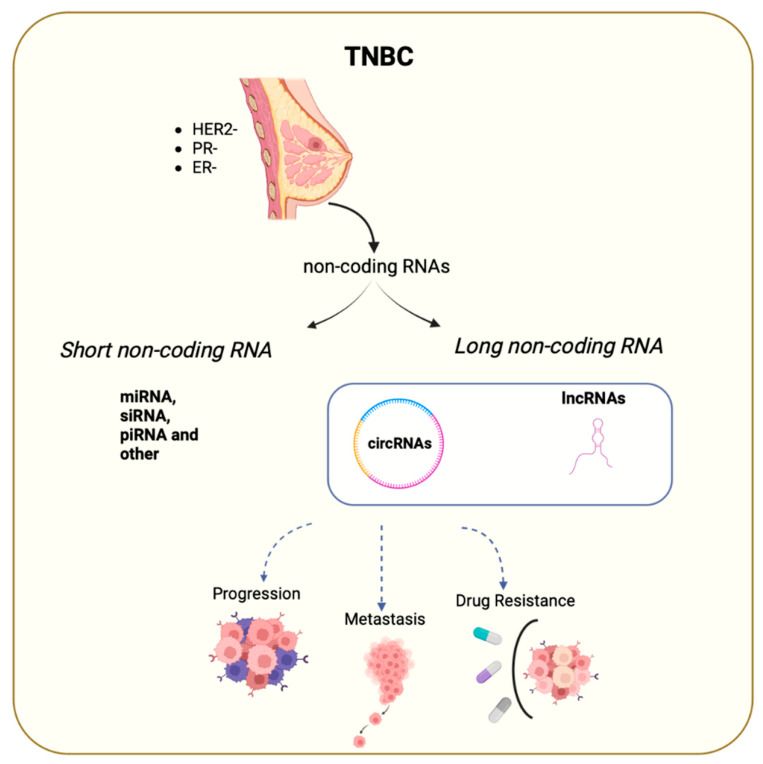
TNBC represents the most aggressive breast cancer subtype because it lacks the expression of druggable receptors. In that context, understanding the role of non-coding RNAs could represent an opportunity for the development of new therapeutic strategies. Short (miRNA, siRNA, piRNA) and long non-coding RNA (circRNA, lncRNA) have been found to modulate TNBC tumor growth and progression and to promote the metastatic process, as well as drug resistance.

**Figure 2:**
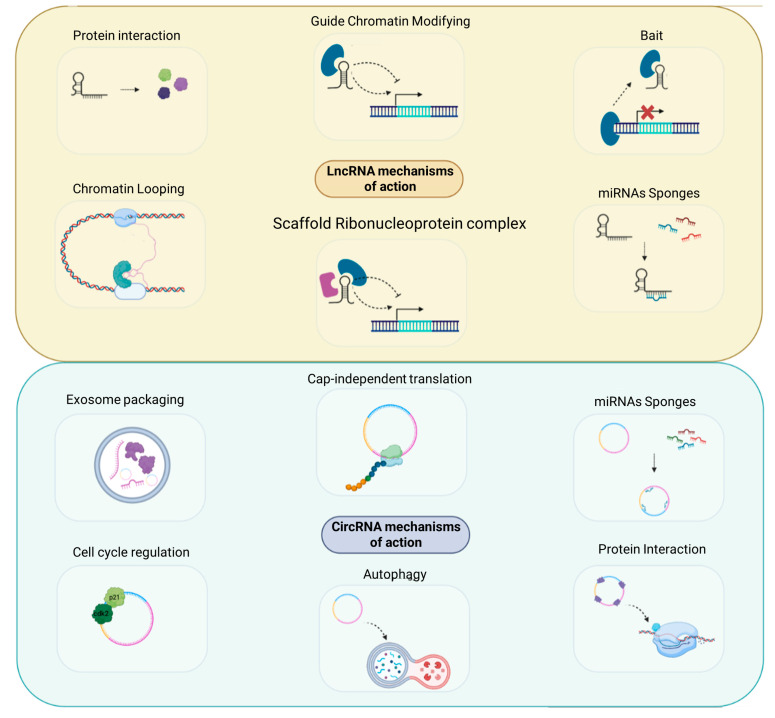
Long non-coding and circular RNA main mechanisms of action. LncRNAs can inhibit or promote gene transcription by creating RNA-DNA duplex, by interacting with proteins, by promoting the formation of scaffold ribonucleoprotein complex, or by remodeling chromatin structure. They can restore protein translation by sponging miRNAs. Similarly, also circRNA can sponge miRNAs or interact with proteins. circRNAs and lncRNAs can encode for peptides.

The ENCODE project revealed that 98% of the transcribed part of the human genome gives different RNAs that do not encode for proteins. This class of RNAs is called protein non-coding RNAs (ncRNAs), and in the last years, they have been intensively studied because of their pivotal role as gene regulators, participating in several cellular processes and also in cancer. Indeed, ncRNAs dysregulated expression is often associated with tumor progression and metastases. Among the different ncRNAs classes, long non-coding RNAs (lncRNAs) and circular RNAs (circRNAs) exert an important role in TNBC (Figure 1). LncRNAs are non-coding RNA molecules, defined as transcripts with more than 200 nt that are transcribed by RNA Polymerase II. Based on their transcription pattern, lncRNAs can be classified as intergenic, exonic, intronic, sense, antisense, or bidirectional. Similarly, to messenger RNAs (mRNAs), pre-mature lncRNAs undergo 5′capping with methyl-guanosine and 3′ polyadenylation and can also be subjected to RNA-splicing. Most of the lncRNAs undergo alternative splicing, which increases the diversity of isoforms produced and subsequently their subcellular localization [7]. LncRNAs functions are based on their nuclear localization, as proven by the fact that they can act as cis or trans RNA regulators (Figure 2). They can inhibit gene transcription by creating an RNA-DNA duplex preventing RNA Polymerase II activity or can mediate chromatin methylation through the recruitment of the Polycomb Complex [8]. Moreover, they can act as a scaffold, bringing together proteins to favor their direct interaction, such as lncRNA TERRA that participate in the telomerase modulation activity [9]. 

They can also act as a decoy or as a guide, thus directing particular ribonucleoprotein complexes to their specific targets. Moreover, lncRNAs can exert sponge activity on particular microRNAs (miRNAs), preventing their inhibitory function activity on their mRNA targets [10] (Figure 2). In addition to the above-mentioned functions, recent studies revealed the involvement of lncRNAs in viral infections [11], as well as a direct interaction with signaling receptors [12].

Deregulated expression of lncRNAs has been associated with different diseases, including cancer [13]. Based on their activity, lncRNAs can be identified as tumor suppressors or oncogenes in cancer. Frequently in cancer, tumor suppressors lncRNA appeared to be non-functional, leading to the activation of oncogenic pathways, whereas the oncogenic lncRNAs are highly expressed, which sustains tumor development and progression. Moreover, their levels can be used as predictive biomarkers for treatment efficiency since their levels can be associated also with tumor recurrence and metastases. Drug resistance is the main reason for therapy failure. Subsequently, lncRNAs levels can be exploited in order to monitor therapy advancement. 

Circular RNAs (circRNAs) are a class of ncRNAs that present a peculiar covalently closed structure. 

Initially considered as splicing errors, later it has been assessed their pivotal regulatory roles in cells [14]. They derived mostly from a process called back-splicing, by which a downstream 3′ splice site is joined to an upstream 5′ splice site, resulting in a closed loop-like structure. The absence of 3′ or 5′ polarity allows circRNAs to be resistant to the action of exonucleases, as shown after RNase R treatment that leads to circRNAs enrichment. Regarding the circularization process, it is favored by the presence of complementary Alu elements in the flanking intronic regions [15]. In addition, RNA binding proteins can be involved as FUS and Quaking (QKI), which have been demonstrated to promote the RNA circularization process [15,16]. CircRNAs mechanisms of action include (1) interaction with proteins acting as a scaffold to promote proteins or RNA Polymerase II binding; (2) regulation of processes, such as autophagy or cell cycle; (3) cap-independent translation leading to the formation of small functional peptides; and (4) sponge activity on proteins or miRNAs (Figure 2). In particular, they can bind their miRNAs targets, preventing their inhibitory activity on the mRNAs targets that result in being upregulated. As in the case of lncRNAs, also circular RNAs exert pivotal roles in cancer, acting as oncogenes or oncosuppressors. They participate in tumor progression, can promote metastases, and can be associated with drug resistance. Moreover, circulating circRNAs that can be found in fluid liquids, such as blood or saliva, have opened new possibilities to exploit these molecules as molecular biomarkers, to monitor therapy treatment, and to predict possible tumor recurrence. Liquid biopsy, which is a powerful tool based on the evaluation of circulating tumor DNA or tumor cells, can be exploited for early diagnosis, prognosis, and to monitor the response to treatment. 

Intriguingly, recent evidence revealed that long non-coding and circular RNAs can encode for peptides (less than 100 amino acids) or proteins [17]. Although their activities, as well as their mechanism of encoding, have to be further elucidated, they represent promising targets for the development of new drugs. As their non-coding counterpart, indeed, they have been found to modulate tumorigenesis. 

Here, we will describe the role of the so-far investigated lncRNAs and circRNAs involved in triple-negative breast cancer pathogenesis, depicting their role as oncogenes or tumor suppressors and highlighting their clinical relevance.

## 2. Long Non-Coding RNAs in TNBC

The study of differentially expressed lncRNAs in TNBC could be useful to assess their involvement in tumorigenesis. Even though the number of the published papers concerning the oncogenic or oncosuppressor role of lncRNAs and their use as molecular targets for TNBC increased exponentially in the last few years, further studies are needed in order to clarify the exact mechanisms they go through.

Several lncRNAs are reported to act as oncogenes in TNBC and also to correlate with tumorigenic features, such as cell proliferation, invasion, migration, and drug resistance. LncANRIL has been reported to be upregulated in TNBC and serous ovarian cancer [18]. It acts as an oncogene by inhibiting apoptosis trough the sponging of miR-199a [18]. Since lncANRIL has increased plasma levels, it represents a good candidate to serve as a molecular biomarker [19,20]. In addition, lncANRIL is involved in chemotherapy resistance by enhancing aerobic glycolysis, and further studies are needed to understand in which way it could be used as a prognostic predictor and for the monitoring of the treatment [21,22]. Another lncRNA that is related to the TNM stage, lymph node, and distant metastases in TNBC is HIF1A-AS2 [23]. It is reported to be upregulated in plasma and to act as an oncogene by an effect on cell proliferation and invasion [19,24]. Moreover, Jiang Y.Z. and colleagues reported that HIF1A-AS2 is associated with paclitaxel resistance, highlighting that this lncRNA could be used as a target for future treatment [25]. The peculiar role of lncRNA downregulated in triple-negative breast cancer is related to lnc00056, an intergenic lncRNA that encodes for a micropeptide of 55 kDa called CIP2A binding protein (CIP2A-BP), which has a tumor suppressor role [26]. Intriguingly, Guo B. and colleagues showed that CIP2A-BP downregulated expression correlates with increased invasion and migration in TNBC. Moreover, they showed that TGF- β, through the activation of the Smad signaling pathway, inhibits the translation of CIP2A-BP, thus supporting TNBC tumorigenesis [27]. HOTAIR is a well-studied lncRNA that has been reported to have a peculiar role also in TNBC. Indeed, it is a marker of TNBC metastases and correlated with poor survival and poor response to chemotherapy [28,29,30]. It is involved in the reprograming of the chromatin state [30,31,32,33]. Interestingly, studies by Li Z.X. and Wang Y.L. report that knockdown of lncRNA-HOTAIR could overcome TNBC resistance to doxorubicin through the PI3K/AKT/mTOR pathway. Of note, the combined treatment, by inhibiting EGFR and c-ABL, has been found to inhibit HOTAIR, leading to the suppression of TNBC growth [33,34]. Moreover, Wang Y.L. and colleagues also report that the use of lapatinib and imatinib, an EGFR/HER tyrosine kinase and KIT inhibitor, respectively, transcriptionally represses HOTAIR, highlighting the important impact that this lncRNA has on TNBC [33].

Long intergenic non-coding RNA regulator of reprograming, lincRNA-RoR, is another peculiar lncRNA strongly overexpressed in TNBC, where it exerts oncogenic activity by acting as a competing endogenous RNA sponge. Indeed, Eades G. and colleagues reported that in TNBC, lincRNA-RoR is associated with metastases by downregulating the oncosuppressor miR-145 [35]. Moreover, Hou P. and colleagues described an association between the lncRNA expression with the epithelial-to-mesenchymal transition process in breast cancer [36]. These data were confirmed by Eades G. and colleagues, which demonstrated, in the TNBC context, how miR-145 does not affect cell proliferation or apoptosis, but it is mainly involved in cell invasion [35,36]. Li Y. and colleagues highlighted the importance of lincRNA-RoR inhibition in breast cancer, which leads to autophagy and also is able to reverse the resistance to tamoxifen, thus suggesting the pivotal role of this lncRNA as a therapeutic target in TNBC [37]. 

LncRNA metastasis-associated lung adenocarcinoma transcript 1 (MALAT-1) results in being upregulated in cancer and, in particular, in TNBC, where Zuo Y. and colleagues reported that its expression correlates with increased cell migration and invasion [38]. Moreover, they found that it exerts its oncogenic role by acting as a miRNA sponge for miR-129-5p. Further analysis by Mekky’s group investigated the role of MALAT-1 as an immunomodulatory lncRNA, showing that it mediates the innate and adaptative immune response by acting as a miRNA sponge regulating miR-34a/MICA/B and miR-175p/PD-L1/B7-H4 axes [39]. Interestingly, even though lncRNA DANCR originally was identified as an oncogene in hepatocellular carcinoma, Sha S. and colleagues revealed that DANCR results in being upregulated also in TNBC, where it could represent a promising target for TNBC treatment [40,41]. Indeed, it was found to be correlated with increased cell proliferation and invasion, and also in the maintaining of CSC-like phenotypic traits by regulation of the expression of several stemness-related genes, such as Nanog, SOX2, and OCT4. Wu G. and colleagues found that DANCR regulates SOX2 expression by sponging miR-874-3p, and, more interestingly, they found that the expression of this lncRNA is induced by Tuftelin (TUFT1), which is in turn positively correlated with poor prognosis. This highlighted that a TUFT1-related therapy against DANCR could be useful for TNBC treatment [42]. Intriguingly, the study of Vaidya A.M. and colleagues demonstrated the efficacy of a TNBC therapy based on nanoparticle-mediated RNAi of the oncogenic lncRNA DANCR, in particular, using tumor-targeting RGD-PEG-ECO/siDANCR nanoparticles [43]. Their study showed that basic oncogenic features, such as proliferation and invasion, were inhibited by the RGD-PEG-ECO/siDANCR nanoparticles in two TNBC cells, MDA-MB-231 and BT-549. The efficacy of this therapy was demonstrated also in vivo, where the nanoparticles-mediated inhibition of DANCR resulted in a complete inhibition of tumor proliferation, suggesting a powerful tool to use in TNBC treatment [43]. Further studies on the pharmacokinetics and toxicity of the nanoparticles are still mandatory. 

LncRNA NEAT1 has been found to be upregulated in TNBC, where its expression correlates with cell growth, migration, and invasion. Moreover, it mediated cisplatin/taxol drug resistance, suggesting that NEAT1 downregulation could sensitize cancer cells to this treatment [44]. Wang S. and colleagues reported that MIR100HG presents a nuclear localization, and it is involved in the regulation of the cell cycle by modulating the expression of the p27 gene through the formation of an RNA-DNA triplex structure [45]. Intriguingly, MIR100HG expression levels result in being very high in TNBC patients and positively correlate with a poor prognosis in TNBC. Of note, this long non-coding RNA has been found to be overexpressed only in TNBC and not in other types of cancer, thus, representing an interesting target for TNBC patient treatment [46]. HCP5 is another lncRNA whose oncogenic role has been established in TNBC, where it acts as a miRNA sponge for miR-219a-5p, leading to the upregulation of the miRNA target BIRC3 involved in apoptosis [46,47]. LncRNA TINCR could serve as a novel biomarker for early TNBC diagnosis. It is upregulated in the serum of early TNBC patients compared to early BC patients [48,49]. Zhang M. and colleagues demonstrated that TINCR expression correlates with increased cell proliferation, migration, and invasion of TNBC cells BT-549 and SUM-159PT, and it also interacts with miR-761, supporting its expression, which in turn promotes a metastatic phenotype [48,49]. Moreover, the same group reported that TINCR upregulation increases trastuzumab resistance, highlighting that it could be targeted in novel therapeutic treatment to overcome drug resistance [49]. Lastly, among the different lncRNAs dysregulated in cancer, one of the most peculiar oncogenic lncRNAs is lnc-PVT1, which has been found to be upregulated also in TNBC. It is located in a cancer-associated region at 54 Kb downstream of MYC locus. Intriguingly, lncPVT1 is located predominantly in the nucleus, where it exerts different functions, such as being involved in DNA rearrangement or epigenetic modification by interacting with EZH2 of PCR2 complex, leading to promoter methylation of different genes associated with anti-tumor properties. In particular, Li R. and colleagues described the lncPVT1 roles in different types of cancer, including breast cancer. Tang J. and colleagues showed that lncPVT1 exerts an oncogenic role in TNBC, where it interferes with the beta–catenin signaling pathway by binding KLF5 and increasing its stability, leading to increased tumorigenesis [50,51]. A study of Wang L. and colleagues revealed that the lncPVT1 oncogenic role in TNBC occurs also through p21 regulation [52]. This lncRNA is peculiar because, following circularization of exon 2, it originates the circRNA called circPVT1, which was also reported to act as an oncogene in several types of cancer, such as gastric cancer and head and neck tumors [53]. The two molecules, lncPVT1 and circPVT1, share important pathways, in which they exert a pivotal role as oncogenes; indeed, they are involved in tumor progression, apoptosis, cancer metabolism, and drug resistance [54]. However, these molecules are controlled by two different promoters; a synergistic effect cannot be excluded as also the cooperation with the neighbor c-Myc. Until now, studies reported in the literature showed that circPVT1 exerts oncogenic activity in breast cancer by sponging miR-181a-2-3p and miR-29a-3p, followed by increased invasion and drug resistance and supporting breast cancer progression through the HIF-1α pathway [55,56]. However, because of the limited data available for their role in TNBC, further studies are needed to elucidate the molecular mechanisms and in which way they can be targeted for eventual treatment.

LncRNAs previously described that result in being upregulated in TNBC are considered as oncogenes; however, the literature search identified also many tumor suppressor lncRNAs. This is the case of GAS5, a lncRNA that is downregulated in TNBC through a high methylation pattern at the CpG island in the promoter region [57]. Zheng S. and colleagues demonstrated that lncRNA GAS5 upregulation in TNBC leads to an increase in paclitaxel sensitivity and apoptosis by acting as a miRNA sponge for miR-378a-5p [58]. Another tumor suppressor lncRNA downregulated in TNBC is lncRNA XIST, which is known to be deregulated in different cancers. Intriguingly, Li X. and colleagues demonstrated that overexpression of XIST in the TNBC cells MDA-MB-468 and MDA-MB-231 has an anti-tumor effect, leading to cell-growth inhibition. Indeed, they found that XIST directly interacts with miR-454, increasing E-cadherin expression, thus inhibiting EMT, and also promotes apoptosis [59]. Moreover, the study of Lan F. and colleagues reported that serum exosomal XIST results in being overexpressed in recurrent TNBC patients, where it is associated also with a poor overall survival, pointing out that it could be used as a pivotal non-invasive biomarker in order to predict TNBC recurrence [60]. DRHC is another tumor suppressor lncRNA downregulated in TNBC that could be a good candidate for novel therapy treatment since it has as a target the oncogenic lncRNA HOTAIR. Indeed, Yu F.S. and colleagues revealed that DRHC expression is able to inhibit the cell proliferation of TNBC cells BT-549 and HCC70 [61]. In addition, many lncRNAs have been associated with disease development and progression. Zhang et al. identified that the long non-coding antisense transcript of nicotinamide phosphoribosyltransferase (NAMPT), NAMPT-AS, has increased levels in TNBC, and this is linked to shorter survival, lymph node engagement, long-distant metastasis, and advanced tumor stage. The authors proved that NAMPT-AS/NAMPT interfered with the mTOR pathway, thus accelerating tumor progression, and regulated autophagy in vitro and in vivo, underlining the oncogenic role of NAMPT-AS [62]. Another lncRNA, GATA3-AS1, contributed to TNBC progression through CD8+ T-cells escape, degradation of GATA3 protein, and stabilization of PD-L1 [63]. 

In Table 1 are listed all lncRNAs reported in the literature that are involved in TNBC and characterized as upregulated or downregulated, with the respective targets and their clinical implication. 

## 3. Circular RNAs in TNBC

In recent years, the functions and molecular mechanisms of circular RNAs have been deeply investigated, as well as their involvement in tumorigenesis [127]. Lu L. and colleagues used a bioinformatic tool in order to detect differentially expressed circRNAs in breast cancer, while the group of Coscujuela Tarrero developed a particular tool able to characterize and quantify circRNAs [128,129]. Evidence showed that circRNAs that resulted in being downregulated were associated with an oncosuppressor role, while those overexpressed were considered as oncogenes. Interestingly, Wu L. and colleagues found that circIRAK3 derived from the IRAK3 gene is upregulated in TNBC cells and promotes invasion, migration, and metastasis in vitro and in vivo by acting as a miRNA sponge for miR-3607 [130]. CircEPSTI1 is another circRNA upregulated in TNBC studied by Chen B. and colleagues, who showed that it acts on the miR-4753/6809-BCL11a axis supporting tumorigenesis. They demonstrated that knockdown of this RNA molecule inhibits TNBC cells proliferation and increases apoptosis, suggesting that CircEPSTI1 could be a good candidate for future targeted therapy [69]. Analysis of the He R. group performed in TNBC patients revealed the significant role of circGFRA1, which results in being upregulated and correlated with a poor outcome and poor overall survival [131]. They demonstrated that this circRNA is able to act in the cytoplasm as a sponge for miR-34a, supporting the upregulation of the miRNA target GFRA1, its host gene, that has been already shown to act as an oncogene in breast cancer, regulating migration and invasion [131]. TNBC progression, in particular, proliferation and metastasis, is regulated also by another circRNA called circ-UBAP2 that Wang S. and colleagues showed to be upregulated in TNBC and associated with poor prognosis [132]. Indeed, this RNA molecule inhibits by direct interaction the tumor suppressive activity of miR-661, which in turn leads to upregulation of the oncogene MTA1 [132]. Another circRNA associated with a poor clinical outcome in TNBC is circKIF4A. Intriguingly, Tang H. and colleagues found that this circRNA is upregulated in TNBC and positively correlates with the TNM stage, lymph node metastasis, and also the tumor size, indicating that it could be a promising molecular target in TNBC treatment [133]. They found that the circRNA mainly localized in the cytoplasm exerts sponge activity on miR-375, preventing its activity on KI4A expression, whose levels are upregulated in multiple malignancies, including also breast cancer. Considering this evidence, the circKIF4A/miR-375/KIF4A axis could be targeted in a novel TNBC therapeutic treatment to overcome cancer progression [133]. Of note, circKIF4A has been recently demonstrated to reprogram breast tumor glucose metabolism by sponging miR-335, which in turn modulates the expression of the OCT4/ALDOA (aldolase A)-HK2 (hexokinase 2)/PKM2 (pyruvate kinase M2) axis [134]. A peculiar circRNA in TNBC is circPSMA1, which has been reported to be upregulated in this aggressive subtype of breast cancer. Yang S. and colleagues revealed that this oncogenic circRNA is found also in tumor-derived exosomes, highlighting its importance as a biomarker in liquid biopsy [135]. Interestingly, they found that circPSMA1 acts on Akt1/β-catenin pathways by sponging the miR-637 whose oncosuppressive role has been previously described in other tumors, such as hepatocellular carcinoma and glioma [135,136,137]. Also, circHIF1A has been found to be overexpressed in the plasma of TNBC patients, suggesting it could be a good biomarker candidate for liquid biopsy. Chen T. and colleagues demonstrated the positive feedback loop between circHIF1A and the oncogenic NFIB; indeed, this circRNA supports NFIB upregulation by acting as a sponge for miR-149-5p, leading to the activation of AKT/STAT3 signaling pathways and inhibition of p21 expression [138]. Moreover, they show that NFIB exerts a feedback loop by enhancing the transcription of FUS, which promotes circHIF1A biogenesis [138]. CircWAC results in being highly expressed in TNBC, and intriguingly, it has been reported to play a pivotal role in TNBC chemotherapy resistance [139]. Indeed, Wang L. and colleagues revealed that circWAC regulates paclitaxel resistance through miR-142 regulation, leading to the WWP1 oncogene overexpression that, in turn, activates the PI3K/AKT pathway [139]. CircWAC could be a promising candidate as a biomarker for future TNBC therapies, and its inhibition could overcome paclitaxel resistance. Notably, not all circRNAs are associated with oncogenic properties; indeed, there is also evidence about circRNAs that exert tumor suppressive roles. This is the case of circ-ITCH, which is downregulated in TNBC and correlates with short survival, larger tumor size, and also metastasis and advanced TNM stage. The study of Wang S. T. and colleagues investigated for the first time the oncosuppressor role of this circRNA in TNBC. Intriguingly, they found that overexpression of circ-ITCH could inhibit TNBC proliferation, migration, and invasion by acting on its already known miRNA targets, miR-214 and miR-17. Through this miRNAs sponge activity, circ-ITCH regulates its host gene expression that is involved in canonical Wnt signaling pathway inhibition [140]. Further studies on this circRNA molecule could be beneficial to improve our knowledge in order to use it as a novel biomarker for TNBC. Another interesting tumor suppressor circRNA initially found downregulated in glioma is circFBXW7 [141]. Further analysis by the Ye F. group demonstrated that circFBXW7 exerts its oncosuppresive role also in TNBC, where it results in being downregulated and associated with a poor clinical outcome [142]. Intriguingly, they found that this circRNA is able to inhibit TNBC progression by acting as a sponge for miR-197-3p, which in turn negatively regulates FBXW7 expression [142]. Moreover, it has been shown that this circRNA has the peculiarity of encoding for a FBXW7-185aa protein that is involved in the inhibition of proliferation and migration [141,142]. CircFBXW7 activity on its host gene FBXW7 may represent a significant axis, on which further studies can be made in order to better understand the regulatory mechanism of circFBXW7 in TNBC and how it can be targeted as a novel therapeutic opportunity. The miRNA-sponging function of many circRNAs has been linked to TNBC progression. For example, circRAD18 has binding sequences for miR-208a and miR-3164, which subsequently leads to upregulation of IGF1 and FGF2, promoting TNBC progression. In contrast, circRAD18 knockdown in cell lines and xenograft models induces cell apoptosis and impairs tumor growth, cell proliferation, and migration [143]. Some circRNAs drive TNBC progression through the regulation of genes involved in cancer-associated signaling pathways. Zhang et al. demonstrated that circRNA_069718 causes EMT in TNBC cells and regulates both mRNA and protein levels of β-catenin, c-Myc, and cyclin D1 that participate in the Wnt/β–catenin pathway [144]. Other circular RNAs have been demonstrated to play pivotal roles in TNBC associated with tumorigenic features or not; a complete list can be found in Table 2.

## 4. Discussion

Considering the aggressive behavior of TNBC and the lack of molecular therapeutic targets, the role of non-coding RNAs and their implication in tumorigenesis needs to be deeply investigated since they could be exploited for future therapy strategies. In particular, lncRNAs’ and circRNAs’ importance as regulators of biological functions is already assessed. These molecules have been shown to present a differential expression pattern between tumoral and non-tumoral tissues that can be associated with their oncogenic or tumor suppressive abilities. The study of molecular mechanisms and the interplay with their targets could be useful to design promising therapeutic approaches. It has been reported that upregulated lncRNA can interact with proteins and also can directly regulate transcription, protein stability, or being involved in chromatin remodeling leading to upregulation of oncogenes that support tumorigenesis. On the other hand, other lncRNAs that act as tumor suppressors result in being downregulated in TNBC, highlighting that their ectopic expression could be beneficial for the patients. In recent years, several studies were conducted regarding circRNAs involvement in diseases, including cancer. Their most studied function is the ability to act as miRNAs sponges, but also through other mechanisms, they exert a fundamental role in cancer. Recent evidence, indeed, has pointed out that non-coding RNA can encode for peptides. The latter could represent easily druggable targets compared to the RNA.

Of note, non-coding RNAs, due to their stability in body fluids, represent the best candidates for prognostic biomarkers in liquid biopsy, a non-invasive way through which therapy can be monitored. Moreover, among the different functions that these molecules elicit, particular attention must be paid to their involvement in drug resistance since it is one of the main issues of TNBC treatment that needs to be overcome. In conclusion, further studies need to be conducted on TNBC in other to best investigate novel molecular biomarkers that can be used as therapeutic targets.

The second class of RNAs discussed in the current manuscript, the circRNAs, have been identified in many species due to the advances of the transcriptomic and bioinformatic approaches. Subsequently, circRNAs received a lot of attention due to their pivotal role as gene expression regulators. Their tissue-specific expression and role in several important cancer characteristics, such as initiation, apoptosis, cell proliferation, invasion, and metastasis, make circRNAs suitable molecules with diagnostic and therapeutic significance [179,180]. Subsequently, the dysregulation of circRNAs has been considered as one of the major reasons for the appearance and development of TNBC [181]. In the aspect of TNBC and circRNAs, the main challenge remained the identification of these sequences that are responsible for disease occurrence, as well as for the aggressiveness and the invasion to the other tissues and organs. To date, most of the circRNAs identified have been shown to be involved in chemotherapy resistance and different pathways related to apoptosis, EMT processing, autophagy, and ceRNA regulation. The variety of functions performed by circRNAs inside the cells suggests the great impact they have in the case of dysregulation of the process of carcinogenesis. The common effort among clinicians and scientists is to develop methods for an early, non-invasive TNBC diagnosis. The presence of TNBC-associated circRNAs in exosomes provides an opportunity for early diagnosis and prediction [182]. 

## 5. Conclusions

**Figure 3:**
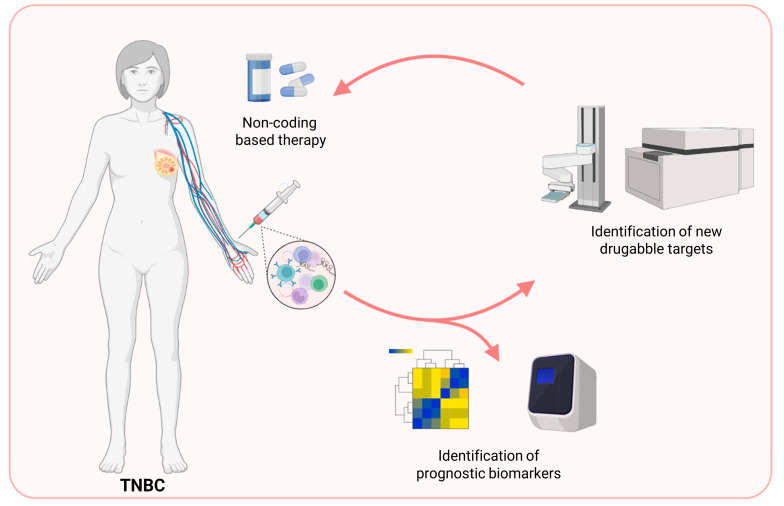
Non-coding RNAs represent an opportunity for TNBC patients’ management. The high-throughput technologies, as well as the high-content screening applications, can favor the identification of new druggable targets and prognostic biomarkers.

Even though both lncRNAs and circRNAs hold great promise not only as molecules for early diagnosis and monitoring, but also for treatment of TNBC, their shift from the laboratory bench to clinical practice still needs to clarify some issues related to their nature. Even though deeply investigated, the exact mechanism by which lncRNAs and circRNAs lead to TNBC development remains elusive. In addition, most lncRNAs and circRNAs investigations are performed on tumor tissues and cell lines. The use of bodily fluids, such as blood, urine, saliva, etc., will open new horizons for TNBC diagnosis and treatment (Figure 3). Finally, the use of lncRNAs and circRNAs as therapeutic targets should be directed to the specific delivery to their needed places for long-term effect and to prevent immune rejection. In summary, additional investigations are required to apply both lncRNAs and circRNAs in current TNBC clinical practice (Figure 3). 

## Figures and Tables

**Table 1:** Comprehensive list of long non-coding RNAs involved in TNBC tumorigenesis.

Name	Function/Target	Biological Effect	Expression Levels		References
			Tumor Tissue	Blood	
ANRIL	Sponges: - mir-199a	Promotes: - cell proliferation, - tumor growth, - chemotherapy resistance, - inhibition of apoptosis.	Upregulated	Upregulated	[16,18,20,21,22]
HIF1A-AS2	NA	Promotes: - cell migration, - cell invasion, - cell proliferation, - paclitaxel resistance.	Upregulated	Upregulated	[19,23,24,25,64]
UCA1	Interacts with: - hnRNP I	Promotes: - cell proliferation, - tumor growth.	Upregulated	Upregulated	[19,65,66]
TINCR	Sponges: - miR-761	Promotes: - cell migration, - cell invasion, - epithelial-to-mesenchymal transition (EMT).	Upregulated	Upregulated	[48,49]
LINC00993	NA	Promotes: - cell cycle arrest, - tumor growth inhibition.	Downregulated	NA	[67,68]
LINC00665	Encodes: - CIP2A-BP micropeptide	Suppresses: - cell migration, - cell invasion.	Downregulated	NA	[26,27]
HOTAIR	Interacts with: - HBXIP - LSD1 Sponges: - mir148a	Promotes: - cell migration, - cell invasion, - drug resistance, - chromatin remodeling.	Upregulated	Upregulated	[28,29,31,32,33,34,69,70,71,72]
lincRNA-RoR	Sponges: - miR-145	Promotes: - cell invasion, - cell migration, - EMT transition, - tumor growth, - drug resistance.	Upregulated	NA	[35,36,37]
MALAT1	Sponges: - miR-182 - miR-129-5p - miR-34a - miR-175p - microRNA-17-92 cluster	Promotes: - cell and tumor growth, - cell invasion, - cell migration, - tumor metastases, - epigenetic modulations. Mediates: - innate and adaptive immune suppression events, - the cytokine storm in tumor microenvironment.	Upregulated	Upregulated	[38,39,73,74,75]
ERRLR01	NA	NA	Upregulated	NA	[76]
LINK-A	Interacts with: - HB-EGF	Promotes: - HIF1α transcriptional activity in normoxic conditions.	Upregulated	NA	[20,77]
LOC554202	NA	NA	Downregulated	NA	[78,79]
LINC01234	Sponges: - miR-429	Promotes: - cell proliferation, - cell migration, - tumor growth.	NA	NA	[80]
lnc-DNAJC16	NA	NA	Upregulated	NA	[81]
lnc-PURA	NA	NA	Upregulated	NA	[81]
CCAT1	Sponges: - miR-218	Promotes: - cell proliferation, - cell migration, - cell invasion.	Upregulated	NA	[82]
TROJAN	Interacts with: - ZMYND8	Promotes: - cell migration, - cell invasion, - tumor growth and metastases.	Upregulated	NA	[83]
LINC00339	Sponges: - miR-377-3p	Promotes: - cell proliferation.	Upregulated	NA	[84]
MIR100HG	Sponges: - miR-5590-3p Determines: formation of RNA–DNA triplex structures through binds to *p27* gene	Promotes: - cell proliferation, - cell migration, - cell invasion, - tumor growth, - drug resistance.	Upregulated	NA	[45,46,85,86]
NRAD1	NA	Promotes: - cell and tumor growth.	Upregulated	NA	[87]
DANCR	Sponges: - miR-874-3p Interacts with: - RXRA	Promotes: - cell proliferation, - cell invasion, - CSC-like phenotypic, - tumor growth.	Upregulated	NA	[40,41,42,43,62,88,89]
NAMPT-AS	Interacts with: - POU2F2 Sponges: - miR-548b-3p	Promotes: - cell proliferation, - cell invasion, - tumor growth, - tumor metastasis.	Upregulated	NA	[62]
Linc-ZNF469-3	Sponges: - miR-574-5p	Promotes: - cell migration, - tumor metastasis.	Upregulated	NA	[90]
HULC	NA	Promotes: - cell migration, - cell invasion.	Upregulated	Upregulated	[23,91]
NEAT1	NA	Promotes: - cell proliferation, - cell invasion, - cell migration, - drug resistance.	Upregulated	Upregulated	[44]
BORG	Interacts with: - TRIM28	Promotes: - drug resistance, - cell proliferation, - tumor metastasis.	NA	NA	[92,93]
HCP5	Sponges: - miR-219a-5p Encodes: - HCP5-132aa protein	Promotes: - cell proliferation, - tumor growth, - drug resistance.	Upregulated	NA	[46,47,94,95]
sONE	Sponges: - eNOS mRNA	Inhibits: - cell proliferation, - cell invasion, - cell migration, - tumor growth, - tumor metastasis.	Downregulated	NA	[96]
PTCSC3	Sponges: - lncRNA H19	Inhibits: - cell proliferation.	Downregulated	Downregulated	[97,98]
NEF	NA	Inhibits: - cell migration, - cell invasion.	NA	Downregulated	[99]
GAS5	Sponges: - miR-378a-5p	Enhances: - drug sensitivity.	NA	NA	[58]
SNHG12	NA	Promotes: - cell proliferation, - cell migration.	Upregulated	NA	[100]
LINC01638	Interacts with: - c-MYC	Promotes: - EMT, - cancer stem cell-like state.	Upregulated	NA	[101]
AFAP1-AS1	Interacts with: - β-catenin	Promotes: - cell proliferation, - cell invasion, - tumor growth, - EMT, - drug resistance.	Upregulated	NA	[102,103]
Linc00152	Interacts with: - DNMTs	Promotes: - cell proliferation, - cell migration, - cell invasion, - tumor growth.	Upregulated	NA	[104,105]
snaR	NA	Promotes: - cell migration, - cell invasion, - tumor growth.	NA	NA	[106]
LncRNA H19	Interacts with: - p53	Promotes: - cell proliferation, - cell migration, - cell invasion, - tumor growth, - drug resistance.	Upregulated	NA	[107,108]
LUCAT1	Sponges: - miR-5702	Promotes: - cell proliferation, - cell migration, - cell invasion, - EMT.	Upregulated	NA	[109]
TINCR	Sponges: - mir-761	Promotes: - cell migration, - cell invasion, - EMT.	NA	Upregulated	[49,85]
lncRNA titin-antisense RNA1 (TTN-AS1)	Sponges: - miR-211-5p	Promotes: - cell proliferation, - cell invasion, - cell migration.	Upregulated	NA	[110]
LINC00299	NA	NA	Upregulated	Hypermethylated	[111,112]
XIST	Sponges: - miR-454 - miR-let-7a-2-3p	Modulates: - cell proliferation, - EMT, - tumor growth, - ALDH+ CSCs.	NA	Upregulated	[59,60,113]
ZEB1-AS1	Interacts with: - ELAVL1	Promotes: - tumor growth, - cell migration, - cell invasion.	Upregulated	NA	[114]
POU3F3	NA	Promotes: - cell proliferation.	Upregulated	Upregulated	[115]
*NRON*	NA	Inhibits: - cell proliferation.	Downregulated	NA	[116]
DRHC	*NA*	Inhibits: - cell proliferation.	Downregulated	NA	[61]
Aim	Activates Wnt/β-catenin/mTOR/PI3K signaling	Inhibits: - cell migration - cell invasion.	Downregulated		[117]
*RMST*	NA	Inhibits: - cell proliferation, - cell invasion, - cell migration.	Downregulated		[118]
PVT1	Interacts with: - KLF5 - Keap 1	Promotes: - cell proliferation, - tumor growth, - drug resistance, - cell migration, - EMT.	Upregulated	Upregulated	[50,51,52,119]
MNX1-AS1	Interacts with: - STAT3	Promotes: - cell proliferation, - cell invasion, - cell migration, - tumor growth.	Upregulated	NA	[120]
AC093850.2	Sponges: - miR-4299	Promotes: - cell proliferation, - cell invasion, - cell migration.	Upregulated	NA	[121]
PRKCQ-AS1	Sponges: - miR-361-5p	Promotes: - drug resistance, - cell proliferation.	NA	NA	[122]
LINC01224	Sponges: - miR-193a-5p/NUP210 mRNA	Promotes: - cell proliferation, - cell migration, - cell invasion.	NA	NA	[123]
LINC01559	Sponges: - miR-370-3p - miR-485-5p - miR-940	Promotes: - cell proliferation, - cell migration, - cell invasion, - tumor growth and metastasis.	Upregulated	NA	[124]
SNHG10	NA	Mediates: - drug resistance.	Downregulated	NA	[125]
LINC00921	Sponges: - miR-9-5p	Inhibits: - cell proliferation, - cell migration, - cell invasion, - EMT.	Downregulated	NA	[126]

**Table 2:** Comprehensive list of circular RNAs involved in TNBC tumorigenesis.

Name	Function/Target	Biological Effect	Expression Levels		References
			Tumor Tissue	Blood	
circIRAK3	Sponges: - miR-3607	Promotes: - cell invasion, - cell migration, - tumor metastasis.	Upregulated	NA	[130]
circKIFI4A	Sponges: - miR-375	Promotes: - cell proliferation, - TNBC progression. Modulates: - glucose metabolism.	Upregulated	NA	[133,134]
circPSMA1	Sponges: - miR-637	Promotes: - cell proliferation, - cell migration, - metastasis.	NA	Upregulated	[135]
circHIF1A	Sponges: - miR-149-5p	Promotes: - cell proliferation, - cell growth, - cell migration, - metastasis.	NA	Upregulated	[138]
circWAC	Sponges: - miR-142	Promotes: - drug resistance.	Upregulated	NA	[139]
circPDCD11	Sponges: - miR-432-5p	Promotes: - aerobic glycolysis, - cell proliferation, - tumor growth.	Upregulated	NA	[145]
circGRAF1	Sponges: - miR-34a	Promotes: - cell proliferation.	Upregulated	NA	[131]
circEPSTI1	Sponges: - miR-4753 - miR-6809	Promotes: - cell proliferation, - tumor growth.	Upregulated	NA	[69]
circUBAP2	Sponges: - miR-661	Promotes: - cell proliferation, - cell migration, - tumor growth, - metastasis.	Upregulated	NA	[132]
circPLK1	Sponges: - miR-296-5p	Promotes: - cell proliferation, - cell migration.	Upregulated	NA	[146]
circRAD18	Sponges: - miR-208a - miR-3164	Promotes: - cell proliferation, - metastasis, - cell growth. Inhibits: - apoptosis.	Upregulated	NA	[143]
circRNA_069718	NA	Promotes: - cell proliferation, - cell migration.	Upregulated	NA	[144]
ciRS-7	Sponges: - miR-1299	Promotes: - cell invasion, - cell migration, - metastasis.	Upregulated	NA	[147]
circITCH	Sponges: - miR-214 - miR-17	Inhibits: - cell proliferation, - cell migration, - cell invasion.	Downregulated	NA	[140]
circTADA2A-E6	Sponges: - miR-203a-3p	Inhibits: - cell proliferation, - cell invasion, - cell migration.	Downregulated	NA	[148]
circFBXW7	Sponges: - miR-197-3p Encodes: - 185-aa protein	Inhibits: - cell proliferation, - cell invasion, - metastasis.	NA	NA	[142]
circANKS1B	Sponges: - miR-148a-3p - miR-152-3p	Promotes: - cancer invasion, - cancer metastasis, - epithelial-to-mesenchymal - transition (EMT).	Upregulated	NA	[149]
circHER2	Encodes: - HER2–103 protein	Promotes: - cell proliferation, - cell invasion, - tumorigenesis.	Upregulated	NA	[150]
circNR3C2	Sponges: - miR-513a-3p	Inhibits: - tumor growth, - metastasis.	Downregulated	NA	[151]
circSEPT9	Sponges: - miR-637	Promotes: - cell proliferation, - cell migration, - cell invasion, - tumor growth, - metastasis.	Upregulated	NA	[152]
circUBE2D2	Sponges: - miR-512-3p	Promotes: - cell proliferation, - cell migration, - cell invasion, - drug resistance.	Upregulated	NA	[153]
circRPH1	Sponges: - miR-195-5p	Promotes: - tumorigenesis, - metastasis.	Upregulated	NA	[154]
circUSP42	NA	Inhibits: - tumor growth.	Downregulated	NA	[155]
circCDYL	Sponges: - miR-190a-3p	Inhibits: - cell proliferation, - cell migration, - cell invasion.	Downregulated	NA	[156]
circZEB1	Sponges: - miR-448	Promotes: - cell proliferation.	Upregulated	NA	[157]
circGNB1	Sponges: - miR-141-5p	Promotes: - cell proliferation, - cell growth, - metastasis.	Upregulated	NA	[158]
circPGAP3	Sponges: - miR-330-3p	Promotes: - cell proliferation, - cell invasion, - tumor growth, - metastasis.	Upregulated	NA	[159]
circAHNAK1	Sponges: - miR-421	Inhibits: - proliferation, - metastasis.	Downregulated	NA	[160]
circAMOTL1	NA	Promotes: - drug resistance.	NA	NA	[161]
circMTO1	Interacts with: - TRAF4	Promotes: - cell proliferation.	Downregulated	NA	[162]
circ_0062558	Sponges: - miR-876-3p	Promotes: - cell proliferation, - cell migration, - cell invasion, - glutamine metabolism, - tumor growth.	Upregulated	NA	[163]
circ_0001925	Sponges: - miR-1299	Promotes: - cell proliferation, - cell metastasis, - angiogenesis.	Upregulated	NA	[164]
circ_0076611	Interacts with: - EIF4B - EIF4G	Modulates: - cell translational rate.	Upregulated	NA	[165]
circCD44	Sponges: - miR-502-5p - Interacts with: - IGF2BP2	Promotes: - cell migration, - cell invasion, - drug resistance.	Upregulated	NA	[166]
circ-EIF6	Encodes: - EIF6-224aa protein	Promotes: - cell migration, - cell invasion, - cell proliferation.	Upregulated	NA	[167]
circ_0091074	Sponges: - miR-1297	Promotes: - cell proliferation.	NA	NA	[168]
circ_0006220	Sponges: - miR 197-5p	Inhibits: - cell proliferation, - cell migration, - cell invasion.	Downregulated	NA	[169]
circ_0041732	Sponges: - miR-149-5p	Promotes: - cell migration, - cell invasion, - cell proliferation.	Upregulated	NA	[170]
circ_0000520	Sponges: - miR-1296	Promotes: - cell migration, - cell invasion, - cell proliferation.	Upregulated	NA	[171]
circ_0131242	Sponges: - miR-2682	Promotes: - cell migration, - cell proliferation.	Upregulated	NA	[172]
circ_0000199	Sponges: - miR-613 - miR-206	Promotes: - cell migration, - cell proliferation, - cell invasion, - drug resistance.	Upregulated	NA	[173]
circ-PDCD11	Sponges: - miR-432-5p	Promotes: - cell proliferation, - tumor growth, - aerobic glycolysis.	Upregulated	NA	[145]
circ-CSNK1G1	Sponges: - miR-28-5p	Promotes: - cell proliferation, - cell migration, - cell invasion, - glycolysis.	Upregulated	NA	[174]
circ_0044234	NA	NA	Downregulated	NA	[175]
circ-TRIO	Sponges: - miR-432-5p	Promotes: - cell proliferation, - cell migration, - cell invasion.	Upregulated	NA	[176]
dirc-PGAP3	Sponges: - miR-330-3p	Promotes: - cell growth, - metastasis.	Upregulated	NA	[159]
circ-UBR5	Sponges: - miR-1179	Promotes: - cell proliferation, - cell migration, - cell invasion, - metastasis.	Upregulated	NA	[177]
circ_102229	Sponges: - miR-152-3p	Promotes: - cell proliferation, - cell migration, - cell invasion.	Upregulated	NA	[178]
